# Linking academic buoyancy and math achievement in secondary school students: Does academic self-efficacy play a role?

**DOI:** 10.1007/s12144-022-03488-y

**Published:** 2022-07-19

**Authors:** Marie Weißenfels, Dana Hoffmann, Laura Dörrenbächer-Ulrich, Franziska Perels

**Affiliations:** Department of Educational Sciences, Saarland University, Campus A4.2, 66123 Saarbruecken, Germany

**Keywords:** Academic buoyancy, Academic self-efficacy, Math achievement, Secondary school students

## Abstract

Academic buoyancy describes the ability to successfully overcome and recover from setbacks in an academic context (e.g., a poor grade, motivational dips, stress due to upcoming performance exams). This day-to-day form of academic resilience has recently been defined in the context of positive psychology. The present study aimed to gain insights into the mechanisms of academic buoyancy by predicting math achievement. Since there is already evidence that this relationship is rather indirect than direct, we were particularly interested in investigating a potential actor of an indirect effect, namely academic self-efficacy. For this purpose, *n* = 974 students at eleven secondary schools in southwestern Germany were surveyed through a questionnaire. The data were analyzed using a latent variable approach. The results of the study show that academic buoyancy is a significant predictor of math achievement and that this relation is explained through academic self-efficacy, even when controlling for gender. Implications for practice and further research are also discussed.

## Introduction

According to the PISA (Programme for International Student Assessment) survey, in 2018 around 21% of 15-year-old students in Germany achieved the two lowest levels of competence in mathematics and reading skills (Reiss et al., 2019). German students are slightly above the average of OECD (Organisation for Economic Co-operation and Development) countries in reading skills and slightly below average in mathematical skills (Reiss et al., 2019). However, the results suggest that many students face academic adversity in their daily school lives (Reiss et al., 2019). Difficulties such as poor grades, competing deadlines, stress due to upcoming exams, and difficulties with individual subjects, affect almost all students (Martin & Marsh, 2008a). As withstanding these adversities is not easy, some students experience persistent long-term stress, fatigue, loss of motivation, and less engagement in class (Martin & Marsh, 2008a). In this context, academic buoyancy describes the ability to “bounce back” from setbacks and overcome academic adversity (Martin & Marsh, 2008a). It can be understood as a form of day-to-day academic resilience that is present in every student but varies in strength (Smith, 2020). Because students constantly face difficulties in their daily school lives, resilience is an essential skill.

Previous research has linked academic buoyancy to various student outcomes such as school satisfaction (Hoferichter et al., 2021), high hope and enjoyment (Hirvonen et al., 2020), measures of achievement (for a review, see Datu & Yang, 2018) and has indeed been shown to buffer the effect of minor adversities on academic achievement (Hoferichter et al., 2021). Everyday academic adversity is less likely to cause a drop in performance for students with strong academic buoyancy (Putwain et al., 2020a, b). However, it remains unclear whether academic buoyancy and achievement are related directly or indirectly via other constructs (e.g., Colmar et al., 2019) and more research is needed to identify possible mechanisms of this relation. Motivational variables have been found to influence academic buoyancy (Aydın & Michou, 2020) and seem to be particularly promising to explain relations between academic buoyancy and academic achievement (e.g., Aydın & Michou, 2020; Datu & Yang, 2021). Datu and Yang (2021) investigated the relation of academic buoyancy with achievement and academic motivational dimensions and found that autonomous motivation mediated the effect of academic buoyancy on academic achievement. However, an essential motivational variable has not been considered so far: academic self-efficacy. According to Social Cognitive Theory, self-efficacy impacts “how much effort will be expended, and how long it will be sustained in the face of obstacles and aversive experiences” (Bandura, 1977, p. 191). Thus, the constructs of buoyancy and self-efficacy show conceptual overlap (Smith, 2020) and academic self-efficacy has as well been shown to be a significant predictor of achievement (Schneider & Preckel, 2017). Therefore, we assume that self-efficacy could play an essential role in the relation between academic buoyancy and achievement and will investigate its indirect effect in this relationship. This investigation thus provides one step further in the analysis of the exact processes that lead to “better” performance. Understanding these processes is very important, as they give us starting points for the appropriate support of students. As mentioned above, there is already evidence of a probable indirect effect of academic buoyancy on achievement and motivational variables seem to play a role (e.g., Colmar et al., 2019). Thus, academic buoyancy interventions aimed at dealing with daily setbacks are certainly helpful but combining it with strategies to promote self-efficacy could then be most promising. This is especially relevant under the assumption that interventions to foster academic self-efficacy have been proven to be very successful (for meta-analysis see Unrau et al., 2018).

Consequently, the present study is one of the first to examine academic buoyancy in a German-speaking country and relate the construct to academic self-efficacy and math achievement. We consider mathematics particularly relevant when it comes to setbacks at school (e.g., Martin & Marsh, 2008a). The study aims to examine the interrelations of these constructs in greater depth and analyze to what extent the relationship between academic buoyancy and achievement is explained via self-efficacy.

### Academic Buoyancy

According to Martin (2013), “Academic buoyancy has been defined as a capacity to overcome setbacks, challenges, and difficulties that are part of everyday academic life” (p. 488). It is oriented toward the framework of positive psychology due to its focus on achieving “psychological growth and improved well-being over time” (Martin & Marsh, 2008a, p. 54). Thus, academic buoyancy refers to how well students can cope with, overcome, and recover from everyday academic adversity (Colmar et al., 2019; Putwain et al., 2020a, b).

In general, research on academic buoyancy is a rather recent phenomenon; the term was first introduced in educational psychology by Martin and Marsh (2008a) and validated based on the multidimensional theory of the Motivation and Engagement Wheel (Martin, 2007). This model summarizes eleven positive and negative factors and forms of motivation and engagement in students at four integrative higher levels: adaptive cognitive dimension (e.g., self-efficacy); adaptive behavioral dimension (e.g., planning); maladaptive behavioral dimension (e.g., self-handicap); and impeding/maladaptive cognitive dimension (e.g., anxiety) (Martin, 2007). The model aims to summarize forms of motivation and engagement in an understandable way so that teachers and students can become aware of their own positive and negative forms of motivation as quickly and easily as possible (Martin, 2007). The negative (impeding/maladaptive) and positive (adaptive) dimensions can also be differentiated according to their valence, allowing qualitative differences between “low-level negative outcomes” and “major negative outcomes” to be recognized (Martin, 2013, p. 496). Academic buoyancy can generally be used to negatively predict “low-level negative outcomes,” which are represented by the impeding/maladaptive cognitive dimensions of anxiety, failure avoidance, and uncertain control on the Motivation and Engagement Wheel (Martin, 2007).

A construct closely related to academic buoyancy is academic resilience, which is more useful for predicting “major negative outcomes” and is negatively associated with the maladaptive behavioral variables self-handicapping and disengagement (Martin, 2007). Because of the conceptual overlap between academic buoyancy and resilience, the following section provides a definition and brief explanation of academic resilience.

#### Distinction of Academic Buoyancy from Academic Resilience

According to Masten et al. (1990), “Resilience refers to the process of, capacity for, or outcome of successful adaptation despite challenging or threatening circumstances” (p. 425). It is the conceptual equivalent of vulnerability and is predicated on it (Lisi, 2020; Masten, 2001). Resilience is viewed as a dynamic, domain-specific, and interactionist variable (Rönnau-Böse & Fröhlich-Gildoff, 2019; Southwick et al., 2014; Ungar, 2012). Consequently, resilience must be regarded as the constantly changing interaction of people and environments in different contexts.

In educational psychology research, academic resilience – representing a subcategory of resilience in the academic context – has been defined as the increased likelihood of success in school and other life achievements despite significant environmental adversity (Wang et al., 1993). Consequently, academic resilience refers to unexpected educational success despite risk factors and vulnerability (Lisi, 2020). Thus, academically resilient students are those who can maintain high achievement motivation and performance even in the face of stressful events and adverse circumstances (Alva, 1991).

Academic buoyancy and academic resilience show a variety of intersections and have a reciprocal relationship, yet they can be clearly differentiated (Martin & Marsh, 2008a; Smith, 2020). The differences between academic buoyancy and academic resilience, “two distinct adversity-related constructs” (Martin, 2013, p. 498), become evident when the following points are considered more closely. While studies on academic resilience tend to look at small samples with specific characteristics (e.g., children growing up in poverty; Overstreet & Braun, 1999), studies on academic buoyancy examine larger samples of students, since academic buoyancy plays a role for all individuals in achievement situations (Martin & Marsh, 2008a). Furthermore, the constructs differ in their temporal dimension: academic buoyancy refers to everyday setbacks, which vary from day to day, such as (single) bad grades, low motivation, and temporary periods of stress (Martin & Marsh, 2008a). Academic resilience, on the other hand, refers to adversities that can be described as acute and chronic, as they usually last for a long time and represent a massive threat to the person’s learning (Martin & Marsh, 2008a). Less extreme circumstances, daily setbacks, and failures in academic contexts are of particular interest when investigating academic buoyancy which we did in the present study.

#### Research on Academic Buoyancy and Student Outcomes

Due to the novelty of the construct, the state of research is still in its beginning stages. However, a few studies have investigated positive outcomes in students which are related to academic buoyancy.

Students with greater academic buoyancy tend to experience higher levels of perceived control over their academic outcomes (Martin & Marsh, 2008a; Collie et al., 2015). Academic buoyancy also predicts several positive factors such as class participation and completion of tasks (Martin & Marsh, 2006, 2008a). Furthermore, academic buoyancy is seen as a specific form of well-being that refers to the academic context (Miller et al., 2013) and is related to higher self-esteem and life satisfaction (Martin et al., 2013a, b). Apart from that, academic buoyancy positively predicts academic achievement (Datu & Yang, 2018; Martin, 2014).

In a recent review, Datu and Yang (2018) argue that there are two ways in which academic buoyancy is positively related to “key academic and psychological outcomes” (p. 209). On the one hand, academic buoyancy shows a positive relationship to adaptive academic functions (Datu & Yang, 2018) such as effective learning strategies (Collie et al., 2017) and higher achievement (Martin, 2014; Miller et al., 2013). Furthermore, positive associations have been found with performance-related variables such as self-regulation (Martin et al., 2013a, b) and behavioral and emotional engagement (Datu et al., 2018).

On the other hand, academic buoyancy is positively related to performance through its negative association with several maladaptive behaviors and outcomes (Datu & Yang, 2018). Thus, previous studies have shown a negative association between academic buoyancy and test anxiety (Putwain et al., 2012, 2015), emotional instability (Martin et al., 2013a, b), and perceived threat (Symes et al., 2015).

In conclusion, academic buoyancy is a crucial predictor for positive academic outcomes, particularly achievement (Martin, 2014; Yun et al., 2018), which is very important for the educational future of students and therefore highly relevant to investigate. The aim of this study is therefore to examine the relationship between academic buoyancy and achievement in more depth.

#### Academic Buoyancy and Academic Achievement

Studies considering academic buoyancy and academic achievement often focus on academic buoyancy as a moderator in relations with achievement. For example, Putwain et al. (2020a, b) found a moderating effect of academic buoyancy between anxiety and test performance. Accordingly, test performance is highest when anxiety is low and academic buoyancy is high (Putwain et al., 2020a, b). In another study, the negative relationship between worrying and performance was found to be moderated by academic buoyancy (Putwain et al., 2016). Thus, the indirect negative relationship between worry and test performance is stronger when academic buoyancy is lower (Putwain et al., 2016).

In contrast, a person-centered approach was adopted by Putwain and Daly (2013), who conducted a cluster analysis. They identified five distinct clusters of students with similar patterns of characteristics regarding academic buoyancy and test anxiety (Putwain & Daly, 2013). These clusters differed with regard to academic achievement: students in clusters with low test anxiety and high academic buoyancy had the highest academic performance (Putwain & Daly, 2013).

The few studies devoted to the direct relationship between academic buoyancy and achievement suggest that academic buoyancy is a significant predictor of achievement, although the strength of the relationships varied. Martin (2014) found a positive relationship with a relatively small effect size, indicating that academic buoyancy is predictive of achievement (*β* = .13). Yun et al. (2018) reported the significant predictive value of achievement by academic buoyancy with high variance explanation (*β* = .27, *R*^*2*^ = .31).

Moreover, Colmar et al. (2019) examined the predictive value of academic buoyancy on performance and additionally considered the extent to which the relationship is mediated by self-concept. The study suggests a relationship between academic buoyancy and academic achievement, mediated by academic self-concept (Colmar et al., 2019). This approach is very interesting, as self-concept has strong conceptual overlap with academic buoyancy and is also relevant to achievement outcomes (e.g., Seaton et al., 2014). Moreover, the authors found gender to be a relevant covariate: male students were significantly more buoyant (Colmar et al., 2019). Collie et al. (2015) followed the same approach and discovered that control (in the sense of attribution theory; see Weiner, 2010) was a mediator of the relationship between academic buoyancy and achievement. The authors emphasized that further studies are needed to investigate the relationship between academic buoyancy and achievement, as mediated by other variables, because the nature of the relationship between academic buoyancy and achievement still lacks clarity.

Thus, we consider constructs that have conceptual overlap with academic buoyancy (i.e., predictors) to be relevant to the relationship between academic buoyancy and achievement and will therefore take a closer look at empirical predictors of academic buoyancy in the following section.

#### Predictors of Academic Buoyancy

Predictors of academic buoyancy are referred to as the 5 C’s in the literature (Martin & Marsh, 2006; Martin et al., 2010; Smith, 2020). These are **c**onfidence, which is mostly assessed via self-efficacy; **c**oordination, which is represented by planning; **c**ommitment, which is assessed via persistence; **c**omposure, which is determined by low anxiety; and lastly **c**ontrol, which is represented by one’s response to uncertainty. The predictive power of the 5C’s has been repeatedly demonstrated in numerous studies (Martin et al., 2010; Martin & Marsh, 2008a). Smith (2020) added a sixth predictor: **c**ommunity, which refers to stable membership in and support from a group.

In a longitudinal study, the stability over time of the predictors of academic buoyancy was investigated (Martin et al., 2010). It was found that academic buoyancy at Measurement Time 1 and Measurement Time 2, which took place one year later, correlated strongly with each other (*r* = .59, *p* < .001) and that the expression of the construct at the earlier time predicted the expression at the later time (*β* = .21) (Martin et al., 2010). The predictors collected at Measurement Time 1 also showed a statistically significant correlation with academic buoyancy at Measurement Time 2: confidence (self-efficacy; *r* = .38, *p* < .001; *β* = .22); coordination (planning; *r* = .32, *p* < .001; *β* = .16); commitment (persistence; *r* = .37, *p* < .001; *β* = .08); composure (anxiety; *r* = −.66, *p* < .001; *β* = −.59); control (control of uncertainty; *r* = −.48, *p* < .001; *β* = −.27) (Martin et al., 2010).

The predictor confidence, measured by self-efficacy, is of particular interest due to its performance-relevant properties and conceptual overlap with academic buoyancy. Moreover, motivational variables have been shown to be very promising in explaining the relationship to academic achievement (e.g., Aydın & Michou, 2020; Datu & Yang, 2021). However, to the best of our knowledge, self-efficacy has not yet been investigated as a mediator in the relationship between academic buoyancy and achievement. We will therefore fill this research gap and take a closer look at academic self-efficacy.

### Academic Self-Efficacy

In his Social Cognitive Theory, Bandura (1989) states that “persons are neither autonomous agents nor simply mechanical conveyers of animating environmental influences. Rather, they make causal contribution to their own motivation and action within a system of triadic reciprocal causation” (p. 1). This triad consists of the actions, the environment, and personal factors, such as self-efficacy beliefs. Self-efficacy refers to “beliefs in one’s capabilities to organize and execute the courses of action required to produce given attainments” (1997, p. 3). Therefore, self-efficacy encompasses a person’s conviction that he or she can successfully cope with difficult situations and challenges on his or her own (Bandura, 1997) and serves as the most central personal factor in the above mentioned triadic interaction by influencing human action through cognitive (e.g., self-aiding thought patterns), motivational (e.g., effort), and affective processes (e.g., stress experience; Bandura, 1989). Bandura emphasizes in numerous works that self-efficacy must always be considered in relation to the context and content of a situation (content and context specificity) since the assessment of one’s own abilities can vary significantly depending on these conditions (Bandura, 1997). Transferred to the educational context, academic self-efficacy has been described as “the confidence or strength of belief that we have in ourselves that we can make our learning happen” (Hattie, 2012, p. 41). It concerns the expectation of competence and handling demands in academic contexts (Jerusalem & Satow, 1999). Academic self-efficacy has a significant positive correlation with general self-efficacy, optimism, and social self-efficacy—which refers to an individual’s perceived ability to initiate and maintain social relationships (Jerusalem & Satow, 1999; Tsai et al., 2017). Moreover, it is relevant to academic achievement, which will be explained in more detail in the following section.

#### Academic Self-Efficacy and Achievement

Since the introduction of the concept of self-efficacy 35 years ago, most studies have found positive associations between self-efficacy and performance (Asakereh & Yousofi, 2018; Bouffard-Bouchard, 1990; Fang, 2014). Studies examining the relationship between self-efficacy and performance often refer to domain-specific forms of self-efficacy, such as academic self-efficacy. In their meta-analysis, Richardson et al. (2012) found—among more than 20 motivational and personality variables—a moderate positive correlation between achievement and academic self-efficacy (*r* = .31). Furthermore, more than 93% of studies report positive correlations between self-efficacy and performance at the interindividual level (Sitzmann & Ely, 2011).

In addition to correlative effects, the prediction of performance by self-efficacy has also been investigated and confirmed by many studies (Özkal, 2019; Phan, 2012; Pietsch et al., 2003). In a meta-analysis examining 105 factors influencing academic performance, self-efficacy emerged as the second strongest predictor (Schneider & Preckel, 2017).

The assessment of one’s chances of success in an upcoming task also has an impact on numerous components of action, such as motivation, emotion, and behavior (Bandura, 1997; Fuchs, 2005) and correlates positively with positive academic emotions, engagement, and satisfaction (Zhen et al., 2017). In addition, self-efficacy influences other determinants of action such as inclinations, perceived opportunities, and goals (Pumptow & Brahm, 2020). Thus, individuals with high self-efficacy work longer and more intensively on solving a task, show more commitment and motivation, and are less frustrated by a possible failure than individuals with low self-efficacy (Çetin & Aşkun, 2018; Jerusalem & Mittag, 1995). These results are partially explained by these individuals’ anticipated satisfaction upon completion, which creates an incentive to perform (Fuchs, 2005).

### Academic Buoyancy and Self-Efficacy

The relationship between academic buoyancy and self-efficacy has been investigated empirically as well as theoretically. Smith (2020) describes similarities and differences between the constructs. The author makes it clear that (academic) self-efficacy, like academic buoyancy, increases the ability to recover from academic setbacks (Smith, 2020). Thus, Smith describes students with higher self-efficacy and academic buoyancy as more persistent, receptive to advice, and more realistic in their goal expectations, so they are more able to cope with failure if a goal is not achieved. In addition to conceptual overlap, Smith (2020) suggests two basic processes that underlie both constructs: the confidence in one’s ability to deal with mistakes on an emotional level and confidence in one’s ability to learn from, recover from, and correct mistakes. The author sees this as the reason why people with a higher level of self-efficacy also have a higher level of academic buoyancy (Smith, 2020).

In addition to these commonalities, there are also differences between the constructs that need to be established. While academic buoyancy refers to overcoming adversities, academic self-efficacy, on the other hand, describes one’s convictions regarding all school events (Bandura, 1997; Fuchs, 2005). When students are confronted with academic adversity, both academic buoyancy and academic self-efficacy play a role, but academic self-efficacy refers to situations other than difficulties, as it comprises one’s general perception of one’s academic capability (Smith, 2020). To give an example, a student believing he or she can recover from a bad grade would make them academically buoyant while one would speak of a student with high self-efficacy if he or she believed to be generally capable of tackling an exam without major issues. The constructs moreover differ in their origins and temporal reference, with academic self-efficacy referring to longer periods of time than academic buoyancy (Collie et al., 2015; Smith, 2020).

Besides theoretical considerations, self-efficacy has been empirically shown to be a significant predictor of academic buoyancy (Martin & Marsh, 2008a; Yun et al., 2018). Furthermore, in a longitudinal study, academic buoyancy was found to be a statistically significant predictor of self-efficacy (Martin et al., 2010). The relationship between academic buoyancy and self-efficacy is therefore assumed to be reciprocal.

For the purpose of this study, we assume that it is more likely that self-efficacy follows academic buoyancy in predicting academic achievement (see Colmar et al., 2019). More precisely, we assume that previous experiences of overcoming academic adversity and emerging from them stronger result in higher levels of academic buoyancy in students, which in turn increases their sense of self-efficacy. They are convinced that they will be able to master future challenges and perform better. The degree of perceived academic buoyancy thus determines the degree of self-efficacy and, subsequently, performance.

### Present Study

Academic buoyancy is a relatively new construct, but it has been shown to predict academic performance significantly (Martin, 2014). However, many studies prove this relation to be rather indirect than direct (e.g., Collie et al., 2015; Colmar et al., 2019). Moreover, academic buoyancy shows conceptual and empirical overlap with the construct of academic self-efficacy, which also predicts academic achievement (Özkal, 2019; Phan, 2012; Pietsch et al., 2003). The constructs—academic buoyancy and self-efficacy—seem to predict each other reciprocally (Martin & Marsh, 2008a; Martin et al., 2010; Yun et al., 2018). In this study, the central research question will be to investigate if the relationship between academic buoyancy and academic achievement is explained through self-efficacy (indirect effect). In doing so, we will contribute to the current research, as the reciprocal relationship of academic buoyancy and self-efficacy and their relation to achievement have not been thoroughly investigated so far and are of great practical importance. Furthermore, the study aims to introduce the construct of academic buoyancy to German-speaking countries. To date, there has been only one study in Germany, which investigated physics students in their first semester and focused on the prediction of dropout or success by academic buoyancy (Neumann et al., 2016).

In the present study, we will focus on students from secondary schools, as we assume that academic problems and difficulties are more evident and persistent in older students than younger students and therefore play a greater role in the upper grades (Coleman & Hagell, 2007). As previous research has shown, achievement-related motivations, beliefs, and affects are highly subject dependent: While a student can show high interest in a subject like English, he or she can show less motivation or anxiety for mathematics-based subjects (Marsh et al., 2002). The same is true for attributions (Vispoel & Austin, 1995) and self-concept (Marsh, 1990). As Martin and Marsh (2008a) emphasize, a “domain-specific approach to the study of academic buoyancy is important” (p. 59). As students show a decline in the valuing of mathematics-based subjects after transition to junior high (Wigfield et al., 1991) and as mathematics is prone to be associated with anxiety (Bessant, 1995), we consider, in line with Martin and Marsh (2008a), mathematics to be particularly relevant when it comes to setbacks at school. Therefore, we will operationalize academic achievement with math grades.

The first hypothesis addresses the relationship between academic buoyancy and academic self-efficacy.

- **H1:** Academic buoyancy is a significant predictor of academic self-efficacy in a linear regression.
- With the second hypothesis, we aim to investigate whether we can replicate the prediction of math achievement by academic self-efficacy.
- **H2:** Academic self-efficacy is a significant predictor of math achievement in a linear regression.
- The third hypothesis aims to replicate the prediction of academic achievement by academic buoyancy.
- **H3:** Academic buoyancy is a significant predictor of math achievement in a linear regression.
- Furthermore, to justify testing a model of indirect effects, we will examine if academic self-efficacy is a predictor of academic achievement using multiple regression analysis with academic buoyancy.
- **H4:** Academic self-efficacy is a significant predictor of academic achievement in a multiple regression with academic buoyancy.

In the final hypothesis, we will test the assumption that the relationship between academic buoyancy and math achievement is mediated by self-efficacy. In line with Colmar et al. (2019), we will also consider gender as a covariate.

- **H5:** Academic buoyancy has an effect on academic achievement through self-efficacy.

## Methods

### Sample

The sample of this study was nested and consisted of *N* = 974 students (Level 1) of *N* = 11 schools (Level 2) in southwestern Germany (average cluster size, *M* = 88.55). Of the participating students, 50.2% were female. The students were in Grades 5 to 10 and were between 10 and 19 years old (*M* = 12.99, *SD* = 1.86). According to Fritz and MacKinnon (2007), the sample size for a mediation analysis using bias-corrected bootstrapping should be at least *n* = 462.

No outliers were identified (± 3SD). We had 3.59% missing values in our variables but no systematic patterns of missing data. To analyze if the missing values were missing completely at random (MCAR), we conducted Little’s (1988) MCAR test which resulted in a non-significant output (*χ*^*2*^ = 402.73, *df* = 382, *p* = .223). As this indicated our missing data to be MCAR, we decided to apply the recommended full information maximum likelihood (FIML) estimations (Enders & Bandalos, 2001).

### Procedure

Data were collected from October to December 2019 in schools whose principals had agreed to participate in the study. Prior to data acquisition, declarations of consent were distributed in class. Participation was voluntary and only those students who could provide a signed consent form from a parent or guardian were allowed to participate in the study. Teachers received the questionnaires in advance, with detailed instructions to ensure objectivity of implementation. Questionnaires were then completed in class. First, students provided information about their gender, age, grade level, and their most recent report card grade in mathematics. School grades were given according to a 15-point system, with 15 points representing the best possible grade (“*very good*”) and 0 points representing the worst grade (“*insufficient*”). The reported grades in mathematics, which were used as a performance measure, had an average of *M* = 8.86 points (*SD* = 2.94). Finally, students completed the items on self-efficacy and academic buoyancy.

Data collection was in line with the ethical standards of the Ethics Committee of the Faculty for Empirical Human Sciences and Economical Sciences (Saarland University) and the data protection committee of the Ministry of Education in Saarland. All data were handled anonymously.

### Instruments

The following section presents the instruments used to collect the data on the relevant constructs. To answer the questionnaire items, students were presented a six-point Likert scale to indicate how strongly they agreed with the given statements. The possible answers ranged from 1 *(“strongly agree)* to 6 (*“strongly disagree”)*. For better understanding, items were recoded so that high scores indicated high expressions of the relevant factor. Scale means were calculated for the questionnaires. To determine internal consistency, Cronbach’s alpha was calculated for all scales.

#### Academic Self-Efficacy

To assess students’ academic self-efficacy, we slightly adapted an instrument by Schwarzer and Jerusalem (1999) comprising six items by replacing words that are no longer used today with more common words in order to make the items understandable for all students. Internal consistency was satisfactory (α = .84). Moreover, to calculate a latent mediation model, we first tested the factorial structure of the instrument with help of a confirmatory factor analysis (CFA) modeling the six items as manifest variables and academic self-efficacy as latent first-order variable. Model fit indices were used to evaluate the goodness of model fit (see Data Analysis Section for more information). The CFA for academic self-efficacy yielded satisfactory results with RMSEA = .05; SRMR = .03; CFI = .97 and *χ*^*2*^*/df*-ratio = 3.76.

#### Academic Buoyancy

For academic buoyancy, we used a translated version of a common questionnaire by Martin and Marsh (2008a, b). The scale consisted of four items referring to subject–independent academic buoyancy (e.g., “I’m good at dealing with setbacks – e.g., bad marks, negative feedback on my work.”). Again, internal consistency was satisfactory (α = .80) and the CFA for academic buoyancy as latent first-order factor with four manifest variables resulted in an excellent model fit with RMSEA = .05; SRMR = .02; CFI = .99 and *χ*^*2*^*/df*-ratio = 3.05.

#### Math Achievement

In addition to questionnaire items regarding academic self-efficacy and academic buoyancy, students’ most recent report card grades in mathematics were collected, ranging from 0 points (*“insufficient”*) to 15 points (*“very good”)*.

### Data Analysis

Analyzing our data, we followed a latent-variable approach due to the advantages of SEM. To do so, we used the software MPlus8 (Muthén & Muthén, 2012) with FIML estimations. Having a nested data structure, we first used the “twolevel” approach in MPlus to estimate an empty model with the intraclass-correlation (ICC), the proportion of variance that can be attributed to the school level (Field et al., 2012). In doing so, we found a very small ICC, according to Arend and Schäfer (2019) for our dependent variable (ICC = .021). This means that only about 2% of the variance within math achievement can be attributed to the different schools. The small ICC—along with the recommendation not to conduct multilevel analyses with fewer than 50 clusters due to estimation bias (Maas & Hox, 2005) and the inability to include bootstrapping estimations with the “twolevel” or “complex” approach in MPlus—led us to decide against a multilevel approach for our data.

**Fig. 1:**
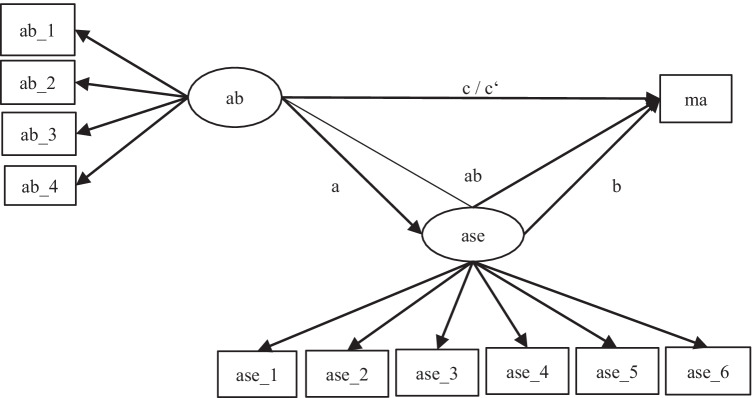
Final mediation model controlling for gender. *Note*. ab = academic buoyancy; ase = academic self-efficacy; ma = math achievement; ab = indirect path; c’ = total path

Thus, we designed our models step-by-step according to our hypotheses as well as the four steps of mediation analysis (Baron & Kenny, 1986). The variables used were academic buoyancy, academic self-efficacy, and math report card grade. As prior research has emphasized the relevance of gender to academic buoyancy (e.g., Colmar et al., 2019), we included gender as a covariate in the final model. All analyses were conducted based on latent variables. In the first step, we tested if academic self-efficacy could be predicted by academic buoyancy (H1, path a). In the next step, we regressed math achievement on academic self-efficacy (H2, path b) and academic buoyancy (H3, path c). Then we employed a multiple regression with academic buoyancy and academic self-efficacy as predictors for math achievement, to see if academic self-efficacy remained a significant predictor and could be used in a mediation model (H4, path c’/total path). The final mediation model included all previously mentioned relations and the indirect path from academic buoyancy to math achievement via academic self-efficacy (H5, path ab). Finally, we included gender as a covariate. The final mediation model is depicted in Fig. 1.

Model fit was determined according to common recommendations (Schermelleh-Engel et al., 2003) by means of root mean square error (RMSEA), standardized root mean square residual (SRMR), *χ*^*2*^*/df*-ratio, and a comparative fit index (CFI). RMSEA and SRMR usually vary between 0 and 1 and excellent model fit is indicated with values of .05 or less. The *χ*^*2*^*/df*-ratio should be below 3 for perfect model fit. CFI, taking values from 0 to 1, should be .95 or greater for excellent fit of the data to the hypothesized model.

To test the indirect effect in our mediation model, we included bias-corrected bootstrapping with *n* = 1000 samples to obtain confidence intervals (CI), as recommended in the common literature (Preacher & Hayes, 2004). The indirect path is significant if the CI does not include zero.

## Results

Before addressing our hypotheses, Table 1 gives an overview of the means, standard deviations, and interrelations of all manifest variables that were included in this study.

**Table 1:** Means, standard deviations, and correlations among all scales

	1. AB	2. ASE	3. PER
*M*	4.41	4.47	8.86
*SD*	1.19	.98	2.94
1.	1	.48**	.12**
2.		1	.38**
3.			1

*ab* academic buoyancy, *ase* academic self-efficacy, *per* performance (based on the most recent grade in mathematics)

**p < .01

To test Hypothesis 1 (path a), we used a simple latent regression model with academic buoyancy predicting academic self-efficacy. The model showed acceptable fit with CFI = .96, RMSEA = .06, SRMR = .04, and *χ*^*2*^*/df*-ratio = 4.57. The hypothesized path was significant (*β* = .62, *p* < .001), indicating that academic buoyancy significantly predicts academic self-efficacy in students. The higher the students’ academic buoyancy, the higher their self-efficacy.

For Hypothesis 2 (path b), we tested the path from academic self-efficacy to math achievement, which was significant (*β* = .57, *p* < .001). The model for this hypothesis showed good fit to the data (CFI = .96, RMSEA = .06, SRMR = .04, and *χ*^*2*^*/df*-ratio = 4.44). The greater the students’ self-efficacy, the higher their math grade.

For Hypothesis 3 (path c), we tested the effect of academic buoyancy on math achievement. This model had acceptable fit (CFI = .98, RMSEA = .07, SRMR = .03, and *χ*^*2*^*/df*-ratio = 5.20) and resulted in a significant regression (*β* = .13, *p* = .010). The more buoyant the students, the better their math grade.

In order to estimate the model for Hypothesis 4 (path c’), we conducted a multiple latent regression with the predictors—academic buoyancy and academic self-efficacy—regressed on the dependent variable, math achievement. This model showed good model fit (CFI = .96, RMSEA = .06, SRMR = .04, and *χ*^*2*^*/df*-ratio = 4.02). Academic self-efficacy was a significant predictor in the multiple regression with academic buoyancy (*β* = .47, *p* < .001) which justified testing the mediation model in the next step.

For the final mediation model, we estimated a model with academic buoyancy predicting math achievement *and* academic self-efficacy, as well as an indirect path wherein academic buoyancy predicted math achievement via academic self-efficacy. The model fitted the data to our satisfaction with a CFI = .96, RMSEA = .06, SRMR = .04, and a *χ*^*2*^*/df*-ratio = 4.73. The indirect effect of academic buoyancy on math achievement (path ab) via self-efficacy was significant (*β* = .27, *p* < .001); the 99% CI with bias-corrected bootstrapping did not include zero [0.57–1.33]. In the multiple regression with academic self-efficacy and academic buoyancy predicting achievement (path c’), academic buoyancy was not a significant predictor, which suggests that academic self-efficacy completely mediates the relationship between academic buoyancy and achievement (*β* = −.12, *p* = .060).

**Fig. 2:**
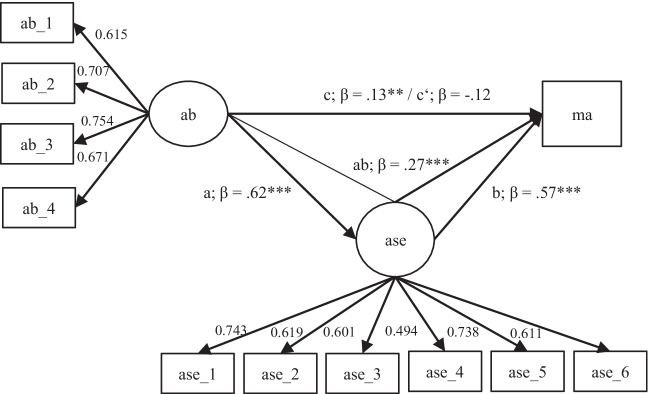
Coefficients of the final mediation model. *Note*. ab = academic buoyancy; ase = academic self-efficacy; ma = math achievement. *** p < .001

Finally, we included gender as covariate and the resulting model fitted the data to our satisfaction with a CFI = .94, RMSEA = .06, SRMR = .04, and *χ*^*2*^*/df*-ratio = 4.22. The indirect effect of academic buoyancy on math achievement via self-efficacy was again significant (path ab; *β* = .27, *p* < .001) and the 99% confidence interval with bias-corrected bootstrapping did not include zero [0.52–1.37]. In this case, academic buoyancy was also not a significant predictor in the multiple regression, which further indicates that academic self-efficacy completely mediates the relationship (path c’; *β* = −.12, *p* = .060). Moreover, correlations with the covariate gender indicated that male students (male coded with 2; female with 1) were, on average, significantly more buoyant (*r* = .07, *p* < .001) but did not experience more self-efficacy (*r* = −.00, *p* = .797) nor were they higher achieving in math (*r* = .01, *p* = .799) (Fig. 2).

## Discussion

The present study was designed to shed more light on the relationship between academic buoyancy, academic self-efficacy, and achievement. In the following section, the results described above are discussed critically, referring to the current state of research. In addition, limitations of the study and implications for future research are provided.

To test our first hypothesis, we investigated whether academic buoyancy is a predictor of academic self-efficacy and found a significant result (*β* = .62). This is in line with prior research (e.g., Martin et al., 2010). Even if a reciprocal relationship is likely, for the purpose of our study, we were particularly interested in the predictive effect of academic buoyancy on self-efficacy, not vice versa.

The significant predictive power of academic buoyancy on achievement, which we investigated in our second hypothesis is also in line with previous findings and shows that academic buoyancy is a performance-relevant construct. These findings are consistent with those of Martin (2014) and Yun et al. (2018). In both studies, the authors found the significant predictive power of academic buoyancy for performance measures. With a regression coefficient of *β* = .13, the results of the present study match the findings of previous studies (Martin, 2014; *β* = .13., Yun et al., 2018; *β* = .27).

To answer our third hypothesis, we further examined whether academic self-efficacy is a significant predictor of math achievement in a multiple regression model with academic buoyancy—if academic self-efficacy predicts variance in math achievement beyond academic buoyancy—and found a significant effect. This justified testing a mediation model. However, we only had one time point at which we measured the predictors as well as the criterium.

The concluding mediation analyses showed that the relationship between academic buoyancy and achievement is fully associated through academic self-efficacy. Thus, when self-efficacy is included in the model, the direct relationship between academic buoyancy and math achievement is not significant. This complete indirect association of the relationship reveals that the performance-relevant effects of academic buoyancy do not directly impact performance. However, this by no means limits the relevance of academic buoyancy to performance. Rather, the results show, especially considering the current research background, that little is known about the mechanisms of academic buoyancy. Previous work, such as that by Colmar et al. (2019) and Collie et al. (2015), provided similar results, involving the mediators of self-concept and control, respectively. The results of the present study thus extend the state of research by considering the influence of a further mediator, self-efficacy. Nevertheless, it has to be mentioned that academic buoyancy and self-efficacy show high interrelation (*r* = .48) and that is why we have to assume a multicollinearity between both variables. This also could cause the decreased relationship of academic buoyancy and achievement in the mediation model. As academic buoyancy and self-efficacy are theoretically linked and interwoven (Smith, 2020), it has to be analyzed in more depth on how to avoid a multicollinearity in future studies.

Moreover, as recommended by Colmar et al. (2019), we accounted for gender as a covariate and considered its relations with the variables in our study. We found no significant correlation between gender and math achievement, which in line with some prior research (Lindberg et al., 2010) but in contrast to other research (Reiss et al., 2019) indicating that male students perform better in math. Moreover, we found academic buoyancy in male students to be higher, which aligns with previous findings (Colmar et al., 2019).

Taking these findings together, we have significantly contributed to the research on academic buoyancy and its relationship with achievement. We have provided another study suggesting that academic buoyancy is crucial to academic achievement, but especially influences other variables that, in turn, impact achievement. Moreover, we replicated previous findings with a large sample allowing structural equation modeling in a German-speaking country where academic buoyancy has rarely been considered thus far.

However, the results of the study should, of course, not be interpreted without considering its limitations.

### Limitations

The fundamental difficulties that can arise from data collection by questionnaire should be pointed out. The questionnaires used to assess academic self-efficacy and academic buoyancy were self-report methods. This means that it cannot be guaranteed that the questionnaire scores given by the students correspond to their actual characteristics in the corresponding constructs. This may be due to the effect of social pressure. It is also conceivable that students did not provide honest information for fear of the information being passed on to the teachers. Nevertheless, there is no appropriate alternative to self-report questionnaires for academic buoyancy or academic self-efficacy, and other studies have implemented them as well.

The cross-sectional design of the study is limiting the implications as well. The project in which the present study took place was initially planned as a long-term study, with two data collection points separated by six months. However, due to the global COVID-19 pandemic, the second measurement point in spring 2020 had to be canceled. Datu and Yang (2018) criticized cross-sectional studies investigating academic buoyancy in their review because no in-depth information can be obtained about how and why academic buoyancy affects performance, for example. Furthermore, calculating a mediation analysis with cross-sectional data is not ideal, as the assumption of the temporal precedence of cause and effect generally underlies all mediation models (Maxwell et al., 2011). It is theorized that the predictor takes place before the mediator, which, in turn, takes place before the criterion. This is not guaranteed with a cross-sectional design. For this reason, some researchers advise not to investigate mediation models with cross-sectional surveys to avoid bias in the results (Maxwell et al., 2011). As already outlined previously, we therefore prefer to speak of an ‘indirect effect’ via self-efficacy. As other studies show, academic buoyancy can also be seen as a mediator between self-efficacy and outcome measures such as test anxiety (Lei et al., 2021). Further studies therefore should aim for longitudinal research on academic buoyancy and self-efficacy so that hypotheses about the directionality of the relation can be generated.

Another limitation is the use of the most recent grade in mathematics as a measure of academic performance. First of all, looking at a single grade on a school report is not sufficiently informative regarding students’ overall academic performance (Südkamp et al., 2012). Mathematics grades, in particular, should be viewed critically due to existing gender stereotypes, which have a negative impact on the mathematical self-concept of female students and can manifest in poorer grades (Steinmayr et al., 2019). For example, in the 2018 PISA study, female students’ math performance was significantly lower than that of male students, which suggests the persistence of gender stereotypes and their influence on performance (Reiss et al., 2019). In contrast, we did not find gender effects on the math grades in our study, which is in line with Colmar et al. (2019). Due to the inconclusive results of previous research regarding the extent to which gender stereotypes and the greater fear of mathematics affect the performance of female students, the exclusive consideration of mathematics performance is clearly limiting. Conducting achievement tests in several subjects to determine the actual performance of students would certainly allow for more informative results.

Further, it could be problematic that the achievement measure was domain-specific and self-efficacy and academic buoyancy were measured domain-general. It is generally assumed that self-efficacy and performance measures correlate especially high when there is the greatest possible correspondence in terms of content (Feng et al., 2015). Various studies discuss that self-efficacy can be based on solving individual concrete tasks (Kürten, 2020). Thus, it is also conceivable to measure self-efficacy by having students solve mathematical problems (Pajares & Miller, 1994) or to measure self-efficacy when learning (Moos & Azevedo, 2008). These assumptions can also be applied to academic buoyancy, which has been measured specifically in some studies (Malmberg et al., 2013). Neumann et al. (2016) assessed academic buoyancy in subject-specific contexts, for mathematics and physics, and described the adaptation as promising. Martin and Marsh (2008b) also adapted the study of buoyancy to a different context—in this case, the workplace—to obtain results that are as close to reality as possible. Future studies therefore should measure self-efficacy and academic buoyancy referring to the same domain as the outcome measure in order to clarify context effects.

### Implications

The results of the present study show that academic buoyancy is a relevant construct that should be further explored in future educational research.

The predictors of academic buoyancy which are referred to as the 5C’s (Martin & Marsh, 2008a) or 6C’s (Smith, 2020) are of great relevance when investigating the role of academic buoyancy in academic performance. However, only a few studies have included predictors or comparable constructs with conceptual overlap with academic buoyancy. Collie et al. (2015) examined the mediating role of control, Colmar et al. (2019) considered self-concept, and the present study focused on self-efficacy. All three studies provide important insights that improve the understanding of the mechanisms of academic buoyancy. However, to gain a complete understanding of the complex interplay between academic buoyancy and other constructs in explaining academic performance, more research is needed.

Moreover, research on academic buoyancy has so far been conducted almost exclusively in industrialized countries, mainly Australia and the UK, and individualistic societies (for a review, see Datu & Yang, 2018). The items assessing academic buoyancy have therefore not yet been validated cross-culturally. As a result, it is unclear how levels of academic buoyancy differ across cultures. Future studies should therefore address the research gap of cross-cultural studies.

Most studies on academic buoyancy have focused on students in higher grades. This focus is justified by the assumption that academic buoyancy plays a more significant role among older students. However, future studies should also look at younger students. As with many educational-psychological constructs with a performance-relevant effect, it can be assumed that academic buoyancy is acquired and trainable in childhood (e.g., self-regulated learning; Dignath et al., 2008).

More research should be dedicated to the questions of *which* students become buoyant and *what* exactly makes them buoyant—for example, by applying person-centered approaches. This could contribute to the development of interventions to make students more resilient to future academic adversity. If an intervention claims to make students more resilient and thus supports them in their everyday school life, academic buoyancy must play an essential role as students do not only suffer from “major negative outcomes,” but also the sum of “low-level negative outcomes” that burden them in the long run (Martin, 2013, p. 496). Some interventions have been successful in building academic buoyancy: For example, a study of Puolakanaho et al. (2019) showed that a five-week online program including information and exercises on psychological flexibility could help improve academic buoyancy. Academic resilience and academic buoyancy would ideally be addressed together in interventions to equip students for all cases of academic and everyday adversity. Besides interventions, strategies that can be taught within the classroom are also possible.

The significant association between academic buoyancy and achievement has been discussed in detail in this paper. However, performance is not the only variable that is positively influenced by academic buoyancy. Miller et al. (2013) addressed the relationship between academic buoyancy and well-being in their work. Following the work of Martin and Marsh (2008a), they suggest that three levels of well-being have a predictive value for academic buoyancy (ibid.). These are psychological factors, school and engagement factors, and family and peer factors (ibid.). Academic buoyancy, in turn, can be used to predict various school factors, such as class participation and task completion (Martin & Marsh, 2006, 2008a). The results of previous studies on students’ well-being and academic performance are sometimes contradictory and lack a theoretical framework to understand the connection (Miller et al., 2013). Miller et al. (2013) suggest that the construct of academic buoyancy may be able to bridge the gap between student well-being and academic achievement. They view academic buoyancy as a possible indicator of general well-being and suggest that academic buoyancy can be predicted not only by school-related predictors, but also by three proximal factors of well-being: psychological factors, school and engagement factors, and family and peer factors. Furthermore, Miller et al. (2013) argue for the promotion of students’ academic performance through academic buoyancy, which they equate with well-being. In general, the consideration of academic buoyancy and well-being reveals that the relevance of the topic is greater than the current state of research indicates so far and further studies are needed to compensate for this deficit.

### Conclusion and Outlook

By examining the relationships between academic buoyancy, academic self-efficacy, and achievement, the present study provided insights into the mechanisms of academic buoyancy. The results are consistent with the previous state of research and extend it in a significant way. However, as academic buoyancy has been little researched so far, further studies dedicated to the construct are urgently needed. With better knowledge, interventions or classroom strategies to support students can be derived. Interventions can be targeted at all students, as the ability to cope with small setbacks is important for all learners. If students are able to recover from the adversities of everyday academic life, this can also be important for later confrontations with greater difficulties. The construct of academic buoyancy should therefore be the subject of greater research interest in the future.

## Data Availability

The datasets generated during and/or analyzed during the current study are available from the corresponding author on reasonable request.
